# Genome-Wide Association for Growth Traits in Canchim Beef Cattle

**DOI:** 10.1371/journal.pone.0094802

**Published:** 2014-04-14

**Authors:** Marcos E. Buzanskas, Daniela A. Grossi, Ricardo V. Ventura, Flávio S. Schenkel, Mehdi Sargolzaei, Sarah L. C. Meirelles, Fabiana B. Mokry, Roberto H. Higa, Maurício A. Mudadu, Marcos V. G. Barbosa. da Silva, Simone C. M. Niciura, Roberto A. A. Torres. Júnior, Maurício M. Alencar, Luciana C. A. Regitano, Danísio P. Munari

**Affiliations:** 1 Departamento de Ciências Exatas, UNESP - Univ Estadual Paulista, Faculdade de Ciências Agrárias e Veterinárias, Jaboticabal, São Paulo, Brazil; 2 Department of Animal and Poultry Science, University of Guelph, Centre for Genetic Improvement of Livestock (CGIL), Guelph, Ontario, Canada; 3 Beef Improvement Opportunities (BIO), Guelph, Ontario, Canada; 4 The Semex Alliance, Guelph, Ontario, Canada; 5 Department of Animal Science, Federal University of Lavras (UFLA), Lavras, Minas Gerais, Brazil; 6 Department of Genetics and Evolution, Federal University of São Carlos (UFSCar), São Carlos, São Paulo, Brazil; 7 Embrapa Southeast Livestock, São Carlos, São Paulo, Brazil; 8 Embrapa Agricultural Informatics, Campinas, São Paulo, Brazil; 9 Embrapa Dairy Cattle, Juiz de Fora, Minas Gerais, Brazil; 10 Embrapa Beef Cattle, Campo Grande, Mato Grosso do Sul, Brazil; Huazhong Agricultural University, China

## Abstract

Studies are being conducted on the applicability of genomic data to improve the accuracy of the selection process in livestock, and genome-wide association studies (GWAS) provide valuable information to enhance the understanding on the genetics of complex traits. The aim of this study was to identify genomic regions and genes that play roles in birth weight (BW), weaning weight adjusted for 210 days of age (WW), and long-yearling weight adjusted for 420 days of age (LYW) in Canchim cattle. GWAS were performed by means of the Generalized Quasi-Likelihood Score (GQLS) method using genotypes from the BovineHD BeadChip and estimated breeding values for BW, WW, and LYW. Data consisted of 285 animals from the Canchim breed and 114 from the MA genetic group (derived from crossings between Charolais sires and ½ Canchim + ½ Zebu dams). After applying a false discovery rate correction at a 10% significance level, a total of 4, 12, and 10 SNPs were significantly associated with BW, WW, and LYW, respectively. These SNPs were surveyed to their corresponding genes or to surrounding genes within a distance of 250 kb. The genes *DPP6* (*dipeptidyl-peptidase 6*) and *CLEC3B* (*C-type lectin domain family 3 member B*) were highlighted, considering its functions on the development of the brain and skeletal system, respectively. The GQLS method identified regions on chromosome associated with birth weight, weaning weight, and long-yearling weight in Canchim and MA animals. New candidate regions for body weight traits were detected and some of them have interesting biological functions, of which most have not been previously reported. The observation of QTL reports for body weight traits, covering areas surrounding the genes (SNPs) herein identified provides more evidence for these associations. Future studies targeting these areas could provide further knowledge to uncover the genetic architecture underlying growth traits in Canchim cattle.

## Introduction

Growth traits are traditionally included in selection criteria in beef cattle breeding programs, due to their association with meat production, and therefore are of great economic importance for both breeders and the industry [1]. The most common type of growth trait used in the selection process is the body weight measurement, which can be taken from birth and throughout an animal’s life. These traits are used not only for evaluation of growth and development, but also for decision making about reproduction, nutritional, and prophylactic management. Body weight usually presents heritability and genetic correlation coefficients from medium to high magnitude [2]–[4].

Technological advances allow the use of massive genotype information through single nucleotide polymorphism (SNP) panels for animal breeding. For cattle, there are several SNP panel densities commercially available from Illumina [5] and Affymetrix [6], varying from a few thousand to more than 3 million SNPs [7], which are applicable for genome-wide association and genomic selection studies. The concept of genomic selection (GS) was first presented by Meuwissen et al. [8], as an alternative to predict more accurate breeding values through genetic markers (i.e. genomic breeding values), and increase the rate of genetic gain by reducing generation intervals. Genome-wide association studies (GWAS) were designed to identify regions that could clarify the inheritance of complex traits [9]. If candidate regions are identified it could be useful for livestock improvement through GS by focusing on relevant genomic regions [10], [11].

For growth traits in crossed beef cattle, important genomic associations for birth weight, weaning weight, and yearling weight were reported by Snelling et al. [12]. These authors also found the same significant SNPs for different growth traits, indicating that a pleiotropic effect exists and genes that are responsible for body weight performance at earlier ages are also acting later on. Furthermore, many of these SNPs observed by Snelling et al. [12] were located in quantitative trait loci (QTL) previously reported for birth weight [13], [14], pre- and post-weaning body weight gain [15], yearling weight [13], stillborn calves [16], and calving difficulty [17]. The presence of QTL for birth weight and calving difficulty support that calf birth weight should be monitored in order to avoid problems with dystocia [18].

Many breeds specialized in meat production are reared in Brazil, such as Canchim, which is derived from crosses between Charolais and Zebu animals. Quantitative research has previously been conducted in this breed to evaluate genetic parameters for growth, reproductive performance, and meat quality traits. However, the specific regions or genes responsible for the genetic variation of these traits are still unknown. Recently, Mokry et al. [19] studied backfat thickness in Canchim cattle and found genomic associations that could aid in GS. Backfat thickness is also a trait of economic importance and has recently gained attention from producers, who wish to increase fat deposition in animals raised on pastures. Thus, the aim of this study was to identify, through GWAS, genomic regions and genes that play roles on birth weight, weaning weight, and long-yearling weight in Canchim cattle.

## Materials and Methods

### Ethics Statement

This study was performed with the approval of the Embrapa Southeast Livestock Ethical Committee of Animal Use (CEUA-CPPSE) under protocol number 02/2009.

### Animals and Data

The BovineHD Beadchip SNP panel from Illumina was used for genotyping 194 males and 205 females, of which 285 were Canchim animals, and 114 were from the “MA” genetic group. The Canchim breed was developed in Brazil through mating schemes between Charolais and Zebu breeds, which generated animals with 5/8 (62.5%) Charolais and 3/8 (37.5%) Zebu fractions [20]. Different schemes were developed to ensure genetic variability and to expand the genetic base of the breed [21]. In particular, the “MA” genetic group produces individuals with an approximate proportion of 65.6% Charolais and 34.4% Zebu, and is very popular among producers. MA animals are the product of crossings between group “A” individuals (derived from crossings between Canchim and Zebu) with Charolais animals [19], [20], [22].

The genotyped individuals were the offspring of 49 sires and 355 dams, born between 1999 and 2005, and originated from seven farms in the states of São Paulo and Goiás in Brazil. In the state of São Paulo, farms were located in the municipality of Águas de Santa Bárbara (22°52′51″S - 49°14′20″W), Angatuba (23°29′24″S - 48°24′46″W), Capão Bonito (24°00′21″S - 48°20′56″W), Sandovalina (22°27′21″S - 51°45′46″W), São Carlos (22°01′04″S - 47°53′27″W), and São Miguel Arcanjo (23°52′40″S - 47°59′49″W). In the Goiás state, animals were from the Jussara municipality (15°51′54″S - 50°52′04″W).

No specific permissions were required for these locations/states and the field studies did not involve endangered or protected species. All farms participate in the Canchim breeding program, in which the database is maintained by Embrapa Beef Cattle through the responsibility of the researchers Dr. Roberto Augusto de Almeida Torres Júnior and Dr. Luiz Otávio Campos da Silva. The genomic data used in this study can be available upon request from Dr. Luciana Correia de Almeida Regitano (Embrapa Southeast Livestock. Address: Rodovia Washington Luiz, km 234, São Carlos, São Paulo, 13560-970, Brazil, Telephone: 55+16 34115600).

Data on estimated breeding values (EBVs) for birth weight (BW), weaning weight adjusted for 210 days of age (WW), and long-yearling weight adjusted for 420 days of age (LYW) were provided by the National Association of Canchim Breeders (ABCCAN) and by the Embrapa-Geneplus Beef Cattle Breeding Program. The EBVs for each trait were obtained by means of multi-trait analysis, which was done using the REMLF90 software [23] under an animal model that included the additive genetic, maternal genetic (only for BW and WW), and residual random effects; contemporary group (CG) as a fixed effect for all the traits, and age of the dam at calving as a covariate. The effects considered in the formation of CGs were sex, birth year and birth season (January to March; April to September; and October to December), farm of birth, genetic groups, and feeding regimen. The relationship matrix of the genotyped animals consisted of a total of 4,095 individuals. The average inbreeding coefficient was 0.02.

### Genotype Quality Control

The SNPs with a genotype calling score lower than 15% were treated as missing genotypes, as recommended by the Infinium platform [24]. Genotype quality control was applied to exclude SNPs with significant (P<10^−5^) Hardy-Weinberg Equilibrium deviation; heterozygous excess (>15%); minor allele frequency (<5%); and call rate (<90%). Animals with call rate lower than 90% were also excluded and only autosomal SNPs with known genome position, according to the UMD_3.1 bovine assembly map [25], were used for the GWAS.

### Genome-wide Association Study

GWAS were carried out by means of the Generalized Quasi-Likelihood Score method (GQLS), developed by Feng et al. [26] and implemented in the SLEUTH software by Dr. Mehdi Sargolzaei. In this method, a logistic regression was used to associate the EBVs (treated as a covariate) with genotypes (treated as a response variable). Analyses were done for one SNP at a time, in which 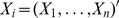 represents the EBVs (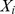) for the *i^th^* animal; and 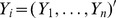 represents the genotypes (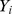), considering 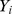 = ½* (number of alleles for the *i^th^* animal’s genotype). As the genotypes were coded as “0”, “1”, and “2”; their respective proportions would be 0, ½, and 1. The expected SNP allele frequency is represented by 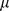, in which 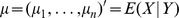, thus 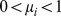.

To associate 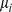 with 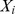, the following logistic regression is defined as:
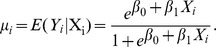
in which *β_0_* is the constant term and *β_1_* the angular coefficient.

The hypothesis to verify the association of the SNP with the EBV assumes:


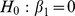
, null hypothesis (the SNP is not associated with the EBV);


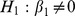
, alternative hypothesis (the SNP is associated with the EBV).

Considering the null hypothesis, 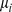 can be interpreted as 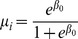, for all 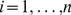. The mean vector of 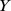 does not depend on 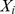, which becomes 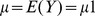, in which “1” is a vector. The solution for the “quasi-likelihood score” equation results in an estimate of 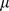. The equation is represented by 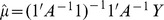, in which 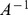 is the inverse of the relationship matrix of all individuals.

To estimate the “generalized quasi-likelihood score”, the 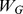 statistics can be calculated by:
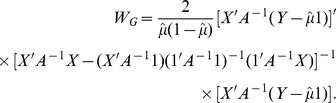



According to Heide [27], under a null hypothesis, 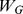 follows a Chi-squared distribution with one degree of freedom, resulting in p-values for each SNP. The advantages of the GQLS method are that it allows an unspecified distribution of EBVs, the method is suitable for studies with either a quantitative trait or a binary trait, and proposes the correction for the population stratification problem by considering the relationship matrix (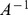) for calculating *W_G_*. However, the effect of the SNP in this methodology is not able to be estimated. Thus, single regression analyses for each significant SNP were carried out, as described in the following section.

### Allele Substitution Effects

Single regression analyses were carried out to verify the genetic additive effects of each significant SNP, previously identified in the GQLS analyses. The following model was applied:
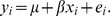
in which 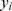 is the EBV for the trait of interest; *µ* is the mean value of the EBV (constant variable);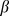 is the linear regression coefficient (allele substitution effect); 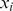 is the number of copies of a given allele (0, 1, or 2); and 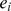 is the residual effect.

### Correction for Multiple Testing

The false discovery rate (FDR) was used for multiple testing correction [28] in order to verify significant SNPs. A maximum threshold of 10% FDR for each chromosome (chromosome-wise) was considered. The p-values of each SNP were sorted in ascending order and the following formula applied:
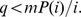
in which *q* is the desired level of significance, *m* is the total number of SNPs, and 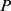 is the p-value of the 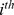 SNP. The SNPs were considered as significant when 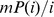 resulted in a value lower than 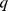.

### Gene Mapping and In-silico Functional Analyses

The significant SNPs were surveyed to their corresponding genes or to surrounding genes within a distance of 250 kb, using the genome databanks National Center for Biotechnology Information (NCBI) [29] and Ensembl Genome Browser [30]. Functional analysis of the mapped genes was performed by means of the UniProt website [31] to verify functional information of the genes.

When no information was available for the *Bos taurus* genes, annotations from human, rat or mouse orthologs were used to proceed with the *in-silico* functional analyses. AnimalQTLdb [32] was accessed to verify previous QTL reported for growth traits in the surrounding regions of significant SNPs.

## Results

The descriptive statistics of estimated breeding values for BW, WW, and LYW are presented in Table 1. As our individuals were a sample from a larger population (of which the breeding values were estimated), it was expected that the mean EBV for each trait was different than zero.

**Table 1:** Descriptive statistics for birth weight (BW), weaning weight (WW), and long-yearling weight (LYW) in animals with genotype information.

Trait	Animals	Mean	Standard-Deviation	Minimum	Maximum
**BW_EBV_ (kg)**	397	0.20	1.34	−4.32	6.15
**WW_EBV_ (kg)**	397	1.24	5.67	−14.87	23.12
**LYW_EBV_ (kg)**	397	0.95	8.98	−24.38	29.24

Estimated breeding values (EBV).

A total of 786,799 SNPs were originally present for each animal, and after the quality control, a total of 672,778 SNPs remained for the GWAS. The number of SNPs evaluated in each *Bos taurus* autosome (BTA) is presented in Table 2. On average, 9.44% of the SNPs were excluded after the genotype quality control. The BTA22 and BTA13 presented the lowest and highest quantity of excluded SNPs, respectively.

**Table 2:** Number of SNPs evaluated for each *Bos taurus* autosome (BTA), BTA length in megabase pair (Mbp), and number of chromosome-wise significant SNPs* for birth weight (BW), weaning weight (WW), and long-yearling weight (LYW), respectively.

BTA	BTA length (Mbp)	Number of SNPs evaluated	BW*	WW*	LYW*
**1**	158.31	42,331	0	0	0
**2**	137.01	36,802	0	0	0
**3**	121.39	32,464	0	0	0
**4**	120.63	32,187	1	1	0
**5**	121.18	31,541	0	0	0
**6**	119.42	32,799	0	2	0
**7**	112.60	30,247	0	0	2
**8**	113.35	30,611	0	0	0
**9**	105.67	28,856	3	0	0
**10**	104.28	28,134	0	0	0
**11**	107.27	29,252	0	9	0
**12**	91.12	23,880	0	0	0
**13**	84.21	21,206	0	0	0
**14**	84.03	22,441	0	0	0
**15**	85.23	22,408	0	0	0
**16**	81.69	22,284	0	0	0
**17**	75.15	20,390	0	0	0
**18**	65.98	17,803	0	0	0
**19**	63.96	17,246	0	0	0
**20**	71.95	19,697	0	0	0
**21**	71.57	19,360	0	0	0
**22**	61.29	16,807	0	0	2
**23**	52.46	13,848	0	0	0
**24**	62.54	17,182	0	0	0
**25**	42.82	11,795	0	0	1
**26**	51.64	13,957	0	0	0
**27**	45.40	11,938	0	0	5
**28**	46.24	12,032	0	0	0
**29**	51.18	13,280	0	0	0
**Total**	**2509.57**	**672,778**	**4**	**12**	**10**

*Total of significant SNPs after multiple testing correction (FDR = 10%).

**Figure 1:**
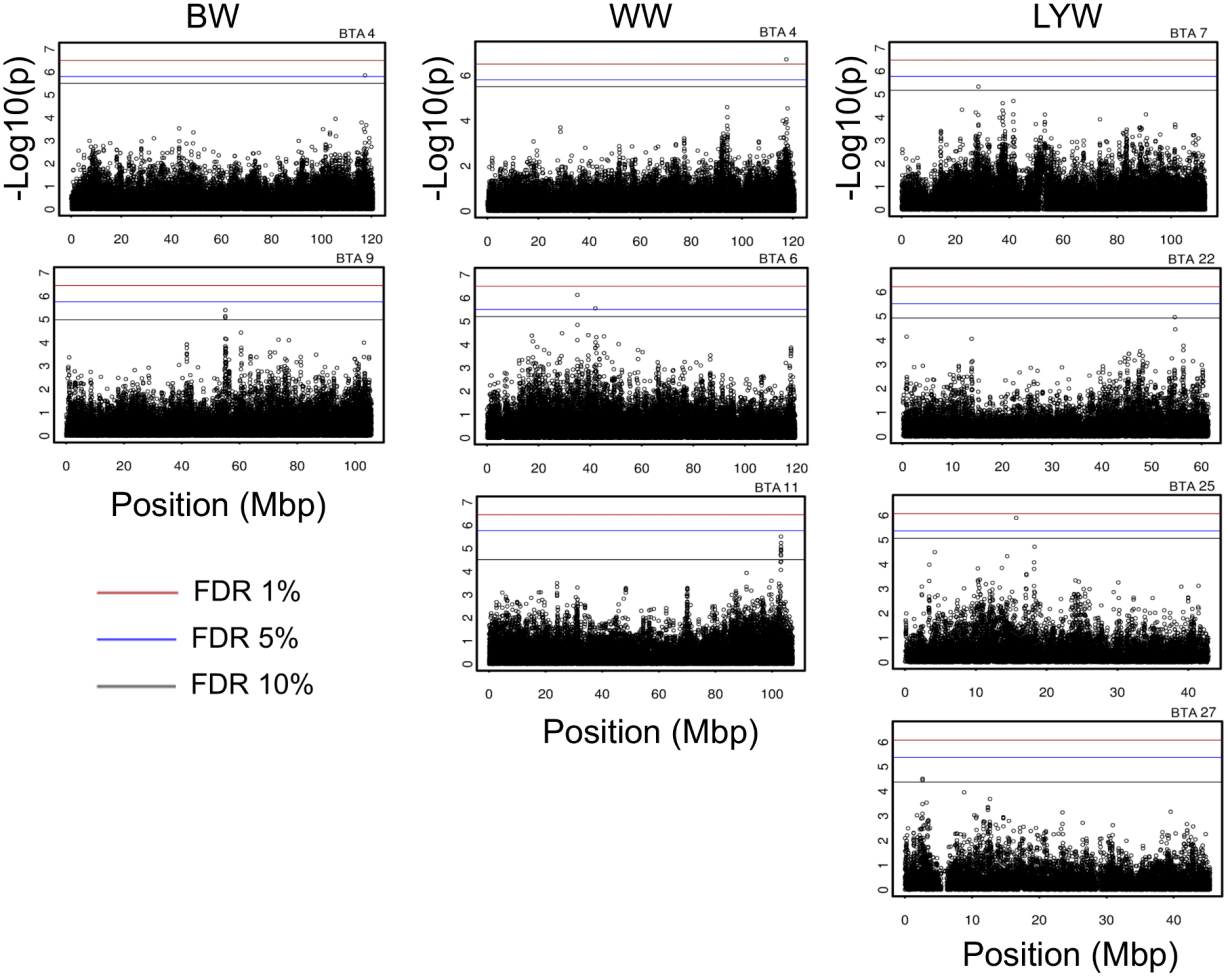
Manhattan plots of p-values for birth weight (BW), weaning weight (WW), and long-yearling weight (LYW). Significance levels were determined by false discovery rate (FDR) correction at levels of 1% (red line), 5% (blue line), and 10% (black line). Positions were presented in megabase pair (Mbp). Associations were observed in autosome (BTA) 4, 6, 7, 9, 11, 22, 25, and 27.

The results from the GQLS and single regression analyses, and SNP and gene identification are presented in Table 3. A total of 4, 12, and 10, SNPs were significantly associated (on a chromosome-wise level) with BW, WW, and LYW, respectively. A total of 18 SNPs were located in gene intron regions, whereas seven were located in intergenic regions, and only one was located in an upstream region. Figure 1 shows the Manhattan plots for chromosome-wise significant regions for BW, WW, and LYW, respectively. Linkage disequilibrium between significant SNPs in each chromosome (BTA) were calculated using the r^2^ coefficient [33], resulting in average values of 0.11 (BTA6, for WW), 0.80 (BTA7, for LYW), 0.91 (BTA9, for BW), 1.00 (BTA11, for WW), 1.00 (BTA22, for LYW), and 1.00 (BTA27, for LYW), respectively.

**Table 3:** Significantly associated SNPs for birth weight (BW), weaning weight (WW), and long-yearling weight (LYW) obtained by the Generalized Quasi-Likelihood Score method (GQLS).

Trait	SNP Reference	BTA	Position (bp)	Alleles	Genes	Region	MAF	p-value	FDR	Allele substitution effect*
**BW**	rs43421095	4	117,400,491	A,C	*DPP6*	Intron	0.2957	1.39E-06	5%	−0.5929
**BW**	rs135754703	9	55,075,535	A,C	*LOC783932, MANEA*	Intergenic	0.4552	8.22E-06	10%	−0.41612
**BW**	rs136146400	9	55,078,557	A,G	*LOC783932, MANEA*	Intergenic	0.4563	7.23E-06	10%	−0.41976
**BW**	rs109313268	9	55,103,057	T,C	*LOC783932, MANEA*	Intergenic	0.4962	3.98E-06	10%	−0.44946
**WW**	rs43421095	4	117,400,491	A,C	*DPP6*	Intron	0.2957	1.91E-07	1%	−2.84898
**WW**	rs135156506	6	35,008,291	T,C	*FARSB*	Upstream	0.1582	7.32E-07	5%	−2.17934
**WW**	rs135591504	6	41,978,318	T,C	*KCNIP4*	Intron	0.2273	2.74E-06	5%	1.55340
**WW**	rs136337296	11	103,096,174	T,C	*GTF3C5*	Intron	0.365	1.81E-05	10%	1.82227
**WW**	rs109348820	11	103,154,983	T,C	*RALGDS*	Intron	0.3916	2.02E-05	10%	1.76133
**WW**	rs133132366	11	103,157,389	T,C	*RALGDS*	Intron	0.4077	1.28E-05	10%	1.81074
**WW**	rs134657108	11	103,162,503	A,G	*RALGDS*	Intron	0.4177	1.26E-05	10%	1.90613
**WW**	rs136054783	11	103,167,055	A,G	*RALGDS*	Intron	0.4066	8.40E-06	10%	1.79279
**WW**	rs136961684	11	103,170,500	A,G	*RALGDS*	Intron	0.4076	1.08E-05	10%	−1.80009
**WW**	rs109945520	11	103,171,584	G,T	*RALGDS*	Intron	0.4047	1.17E-05	10%	1.80350
**WW**	rs109524492	11	103,172,572	C,T	*RALGDS*	Intron	0.4099	5.74E-06	10%	1.63200
**WW**	rs110048168	11	103,174,303	A,C	*RALGDS*	Intron	0.3886	3.05E-06	10%	−1.66682
**LYW**	rs29011435	7	28,515,652	T,C	*MARCH3, LMNB1, PHAX, ALDH7A1,* *C7H5orf48, GRAMD3, MIR2458, LOC100848523*	Intergenic	0.374	4.62E-06	10%	3.59660
**LYW**	rs134201365	7	28,522,539	T,G	*MARCH3, LMNB1, PHAX, ALDH7A1,* *C7H5orf48, GRAMD3, MIR2458, LOC100848523*	Intergenic	0.375	4.62E-06	10%	−3.59660
**LYW**	rs109581958	22	54,624,190	T,C	*LARS2, LOC101907967, TMEM158, LOC101908013, CDCP1, LOC614114, LOC101908094, LOC101901958, ZDHHC3, EXOSC7, LOC100847326, CLEC3B*	Intergenic	0.2335	1.09E-05	10%	−3.61775
**LYW**	rs110246286	22	54,625,467	T,C	*LARS2, LOC101907967, TMEM158, LOC101908013, CDCP1, LOC614114, LOC101908094, LOC101901958, ZDHHC3, EXOSC7, LOC100847326, CLEC3B*	Intergenic	0.2348	1.09E-05	10%	−3.61775
**LYW**	rs109242147	25	15,697,543	A,G	*XYLT1*	Intron	0.1192	1.26E-06	5%	3.24823
**LYW**	rs109646351	27	2,614,991	A,G	*LOC101904868*	Intron	0.4456	3.05E-05	10%	−3.91546
**LYW**	rs109822265	27	2,619,242	A,C	*LOC101904868*	Intron	0.4454	3.05E-05	10%	3.91546
**LYW**	rs110603636	27	2,620,088	A,C	*LOC101904868*	Intron	0.4456	3.05E-05	10%	3.91546
**LYW**	rs110994026	27	2,620,961	T,C	*LOC101904868*	Intron	0.4494	3.46E-05	10%	−3.85715
**LYW**	rs134791735	27	2,623,000	T,G	*LOC101904868*	Intron	0.4443	3.05E-05	10%	3.91546

Single locus regression was carried out to estimate allele substitution effects for significant SNPs.

*Coefficient of regression (P<0.001); FDR  =  False discovery rate; MAF  =  Minor allele frequency; bp  =  base pairs; BTA  =  *Bos taurus* autosome.

## Discussion

### Birth Weight (BW)

The SNP rs43421095 was significantly associated with BW and is located in the *DPP6* (*dipeptidyl-peptidase 6*) gene (Table 3). The *DPP6* gene is involved in proteolysis and nervous system development of mouse [34]. This SNP is located in the same region of a QTL associated with yearling weight and calving ease for Angus cattle [35]. Schrooten et al. [17] observed a QTL associated with gestation length in dairy cattle located between 102.10 and 124.80 cM.

On BTA9, the SNPs rs135754703, rs136146400, and rs109313268 are located close to *MANEA* (*endo-alpha mannosidase*) and the *LOC783932* (*small ubiquitin-related modifier 1-like*) genes. The *MANEA* gene product is related to the glycoprotein endo-alpha-1,2-mannosidase activity, which acts in metabolism of protein pathways [36]. For the *LOC783932* gene, no information regarding its function was observed in the consulted literature. In the same region, Alexander et al. [37] found a QTL associated with post-weaning average daily gain located between 48.73 and 80.26 cM. Snelling et al. [12] observed five significant SNPs (FDR≤10%), located between 112,474,006 and 114,565,961 bp on BTA4; and three SNPs, located between 54,832,703 and 57,515,862 bp on BTA9, associated with birth weight. One of these SNPs found by these authors (rs108988191) is also located close to the *MANEA* and *LOC783932* genes.

For Canchim cattle, Machado et al. [22] found a QTL on BTA5 associated with BW, which contained the microsatellite markers *ILSTS066, TEXAN15,* and *BMS1248* and the microsatellite on the *IGF1* gene. Andrade et al. [20] verified association of BW with microsatellite markers in the *IGF1* gene, also located on BTA5. The reason why we didn’t find associations for BW on chromosome 5 could be due to the differences in experimental designs, and because the previously cited authors studied only animals from the Embrapa Southeast Livestock herd, while in our study animals from other farms and other Brazilian states were used. The genes here identified as associated with BW seem to act on an animal’s body development, with special emphasis on the *DPP6* gene, considering its function in the nervous system. Our discovery also provides more evidences for the genetic relationship of BW with gestation length and calving ease. In addition, our findings corroborate with quantitative genetic studies, which present genetic correlations of moderate to high magnitude between BW and reproductive traits [38], [39].

### Weaning Weight (WW)

On BTA 4, a pleiotropic effect for rs43421095 SNP was found between BW and WW, which corroborates with the results obtained by Snelling et al. [12]. These authors found pleiotropic effects for birth weight, weaning weight, and yearling weight; which means that the genes that are acting on body weight performance at earlier ages are also acting later on. On BTA6, the rs135156506 SNP (Table 3) is located in the upstream region of the *FARSB* gene (*phenylalanyl-tRNA synthetase, beta subunit pseudogene*); which plays a role in aminoacyl-tRNA biosynthesis pathway. For WW, McClure et al. [35] found a QTL on BTA6 between 8.05 and 43.93 cM that contains the rs135156506 SNP. The *KCNIP4* gene (*Kv channel-interacting protein 4*) has a role in the calcium ion binding, and in potassium and voltage-gated ion channel activity [40]. A QTL associated with yearling weight was found by McClure et al. [35] between 46.86 and 53.72 cM.

The *RALGDS* gene product (*ral guanine nucleotide dissociation stimulator*), located on BTA11, is related to the guanyl-nucleotide exchange factor activity and participates in the regulation of small GTPase mediated signal transduction [41], [42]. No biological function for the *GTF3C5* gene (*general transcription factor 3C polypeptide 5*) was found in the literature. Snelling et al. [12] observed three significant SNPs for WW located between 113,506,092 and 114,302,482 bp, on BTA4; five SNPs located between 33,441,527 and 37,584,088 bp, on BTA6; and nine SNPs located between 41,178,449 and 42,609,559 bp, on BTA6. These SNPs were located in similar regions of the SNPs that were significant in the present study. Regarding the Canchim breed, Andrade et al. [20] found associations of WW with microsatellite markers in the *IGF1* gene on BTA5.

### Long-Yearling Weight (LYW)

For LYW, pleiotropic phenomenon was not observed with BW and WW, which means that different genes are acting in earlier and older ages. Significant SNPs close to the *MARCH3* (*membrane-associated ring finger (C3HC4) 3*), *LMNB1* (*lamin B1*), *PHAX* (*phosphorylated adaptor for RNA export*), *ALDH7A1* (*Aldehyde dehydrogenase family 7 member A1*), *C7H5orf48* (*chromosome 7 open reading frame, human C5orf48*), *GRAMD3* (*GRAM domain containing 3*), and *LOC100848523* (*60S ribosomal protein L26-like*) genes and micro RNA MIR2458 (*microRNA mir-2458*) were observed. According to Fukuda et al. [43], the *MARCH3* gene participates in the processes of endocytosis and protein ubiquitination. The LMNB1 gene contributes in the maintenance of structural integrity of a complex or assembly within or outside a cell [44]. The *PHAX* gene acts in protein transport and in snRNA export from the nucleus to the cytoplasm [45]. The *ALDH7A1* gene has a role in the glycine betaine biosynthetic pathway [46]. Information regarding the functions of *C7H5orf48*, *LOC100848523*, *GRAMD3*, and MIR2458 was not found in the consulted literature. Snelling et al. [12] observed a SNP (rs109544319) significantly associated with yearling weight in the *C7H5orf48* gene.

In the region where these significant SNPs were located, no QTL for growth traits has been previously found. However, Sherman et al. [47] found a QTL on BTA7 associated with residual feed intake located between 11.70 and 48.50 cM in Angus, Charolais, and hybrid bulls. Maltecca et al. [48] and Druet et al. [49] verified QTL associated with gestation length and spermatic motility located between 16.70 and 39.50 cM and between 25 and 71 cM, respectively.

SNPs associated with LYW, on BTA22, were located close to *LARS2* (*leucyl-tRNA synthetase 2, mitochondrial*), *TMEM158* (*transmembrane protein 158*), *CDCP1* (*CUB domain containing protein 1*), *LOC101907967* (*uncharacterized LOC101907967*), *LOC614114* (*cytochrome c oxidase subunit VIb polypeptide 1* (*ubiquitous) pseudogene*), *LOC101908013* (*cAMP-regulated phosphoprotein, 19kDa pseudogene*), *ZDHHC3* (*zinc finger, DHHC-type containing 3*), *EXOSC7* (*exosome component 7*), *LOC101908094* (*ribosomal protein L29 pseudogene*), *LOC101901958* (*uncharacterized LOC101901958*), and *CLEC3B* (*C-type lectin domain family 3 member B)* genes. The functions for *CDCP1, TMEM158, LOC101907967, LOC614114, LOC101908013, LOC101908094,* and *LOC101901958* genes were not found in the consulted literature.

The *LARS2* gene is involved in aminoacyl-tRNA editing leucyl-tRNA aminoacylation, and in translational fidelity [50]. The *ZDHHC3* gene product participates in transferase activity (transferring acyl groups) and in interaction with zinc ion [51]. The *EXOSC7* gene participates in positive regulation of cell growth and rRNA processing [52]. The *CLEC3B* gene participates in calcium ion binding. This gene encodes a protein that is important in bone mineralization, cellular response to transforming growth factor beta stimulus, positive regulation of plasminogen activation, and skeletal system development [25], [53]. QTL associated with yearling weight was found by McClure et al. [35], located between 64.08 and 82.93 cM.

The SNP rs109242147 is located in the intron region of the *XYLT1 (xylosyltransferase I)* gene, which participates in the cellular response to heat and in the glycosaminoglycan biosynthetic pathway [54]. McClure et al. [35] observed QTL for yearling weight located between 2.24 and 14.44 cM. On BTA27, five SNPs were significantly associated with LYW and are located in the *LOC101904868* gene (*CUB and sushi domain-containing protein 1-like*). However, no biological functions or QTL were reported for this gene in the consulted literature.

## Conclusions

The Generalized Quasi-Likelihood Score method identified regions on chromosome associated with birth weight, weaning weight, and long-yearling weight in Canchim and MA animals. New candidate regions for body weight traits were detected and some of them have interesting biological functions, of which most have not been previously reported. The observation of QTL reports for body weight traits, covering areas surrounding the genes (SNPs) herein identified provides more evidence for these associations. Future studies targeting these areas could provide further knowledge to uncover the genetic architecture underlying growth traits in Canchim cattle.
